# Intervention of an Upgraded Ventilation System and Effects of the COVID-19 Lockdown on Air Quality at Birmingham New Street Railway Station

**DOI:** 10.3390/ijerph19010575

**Published:** 2022-01-05

**Authors:** Matt Clegg, John Edward Thornes, Debasree Banerjee, Christina Mitsakou, Azhar Quaiyoom, Juana Maria Delgado-Saborit, Revati Phalkey

**Affiliations:** 1 School of Geography, Earth and Environmental Sciences, University of Birmingham, Birmingham B15 2TT, UK; mattclegg210@gmail.com (M.C.); delgado@uji.es (J.M.D.-S.); 2 Climate Change and Health Group, Radiation, Chemical and Environmental Hazards, United Kingdom Health Security Agency, Chilton, Oxford OX11 0RQ, UK; Christina.Mitsakou@phe.gov.uk (C.M.); revati.phalkey@phe.gov.uk (R.P.); 3 Emission Solutions Ltd. (EMSOL), London SEI 0NZ, UK; debasree.banerjee@emsol.io; 4 QSustain Ltd., Stockport SK7 6BG, UK; Azhar.Quaiyoom@networkrail.co.uk; 5 School of Health Science, Universitat Jaume I, 12071 Castellon, Spain

**Keywords:** air pollution, upgraded ventilation system, nitrogen dioxide, particulate matter, carbon monoxide, enclosed railway station, diesel trains

## Abstract

High NO_2_ concentrations (long term average of 383 µg/m^3^ in 2016/2017) recorded at Birmingham New Street railway station have resulted in the upgrade of the bi-directional fan system to aid wind dispersion within the enclosed platform environment. This paper attempts to examine how successful this intervention has been in improving air quality for both passengers and workers within the station. New air pollution data in 2020 has enabled comparisons to the 2016/2017 monitoring campaign revealing a 23–42% decrease in measured NO_2_ concentrations. The new levels of NO_2_ are below the Occupational Health standards but still well above the EU Public Health Standards. This reduction, together with a substantial decrease (up to 81%) in measured Particulate Matter (PM) concentrations, can most likely be attributed to the new fan system effectiveness. Carbon Monoxide levels were well below Occupational and Public Health Standards at all times. The COVID-19 pandemic “initial lockdown” period has also allowed an insight into the resultant air quality at lower rail-traffic intensities, which produced a further reduction in air pollutants, to roughly half the pre-lockdown concentrations. This study shows the scope of improvement that can be achieved through an engineering solution implemented to improve the ventilation system of an enclosed railway station. Further reduction in air pollution would require additional approaches, such as the removal of diesel engine exhaust emissions via the adoption of electric or diesel-electric hybrid powered services.

## 1. Introduction

Before the COVID-19 pandemic, the UK was experiencing increased popularity for rail travel with more than 1700 million passenger journeys per year across the network [1]. Towards the centre of that network lies Birmingham New Street Station, a subterranean, twelve platform interchange, which is the busiest UK station outside of London [2]. In 2016, the station experienced more than 170,000 passengers per day and following redevelopment in 2009–2015, has total capacity for up to 300,000 passengers per day [3,4]. With such a high passenger volume, a correspondingly high rail-traffic volume is found, with more than 1000 trains per day; up to 600 of which are diesel powered [4]. Problematically, the greater length of the 12 platforms resides beneath the ground level concourse area. This results in a low air volume environment (240 m long, 160 m wide and 5 m high) at platform level when compared to other large, enclosed train stations such as London Paddington (250 m long, 100 m wide, and 15 m high) or London St Pancras [5,6]. Due to this low air volume at the platform level, Birmingham New Street station is more like an underground station. These characteristics severely limit the wind dispersion of pollutants within the station, leading to high pollution concentrations at platform level as highlighted in several studies [2,6,7,8]. The highest areas of pollutant concentrations in these studies were at Platforms 10 and 11 where Hickman et al., 2018 [2] found average NO_2_ levels of 383 µg/m^3^ over the period 17 November 2016 until 23 January 2017 with a maximum recorded hourly concentration of 2020 µg/m^3^. Despite the known health (Table 1) and economic implications of air pollution, as well as the plethora of studies on road transport emissions, very few studies [9] have measured air pollution in the context of rail transport in the UK. This study compares the previous monitoring results of 2016/2017 [2] with measurements conducted in 2020 (pre- and post-COVID-19 lockdown) to determine the effectiveness of the 2019 upgraded sensor driven system for the 98 bi-directional fans. The ventilation fans are installed in the roof of the platforms to disperse emissions away from the station. The monitoring campaign in 2016/2017 [2] showed that the original 98 CO_2_ sensors used to trigger the fans were inadequate and Network Rail therefore installed 100 new NO/NO_2_ sensors to help control the ventilation fans instead. Fixed monitoring carried out before and through the initial lockdown for the COVID-19 pandemic has allowed an insight into the success of this upgraded ventilation system in improving air quality within the station.

Table 1 shows the current occupational health and public health limit values for nitrogen dioxide (NO_2_), PM_10_, PM_2.5_, and carbon monoxide (CO) for comparison with the results below.

## 2. Monitoring Air Pollution Levels in the Station

**Figure 1:**
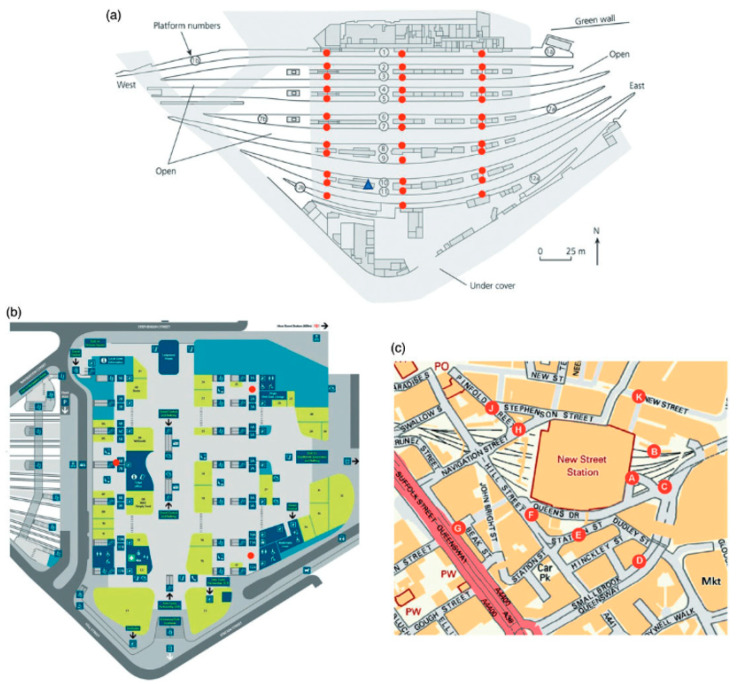
(**a**) A plan view of the below-ground platform layout of New Street Station with a blue triangle indicating the Praxis monitors on Platform 10B and 11B utilised in this study, whilst red dots indicate monitoring sites from the Hickman et al., 2018 study. (**b**) A plan view of the ground-level concourse of New Street Station labelled by usage. Red dots indicating further monitoring sites from the Hickman et al., 2018 study. (**c**) A map of the surrounding area of New Street Station with A–K labelling monitoring sites used as reference values (figure adapted from Hickman et al., 2018 [2]).

Figure 1a displays the subterranean layout of Birmingham New Street’s platforms with curved ends and in the cases of Platforms 8–12 curved centres.

When combined with the low enclosed environment created by the concourse level (marked by grey shading), it becomes apparent that there is limited potential for wind dispersion from outside of the station. With platforms up to 240 m long, it is common for multiple diesel trains to be entirely within the enclosure, highlighting the requirement for the installed bi-directional fan system. Platforms 10 and 11 were noted in the Hickman et al., 2018 study [2] as being the areas of highest pollutant concentrations due to almost entirely diesel-powered services.

**Figure 2:**
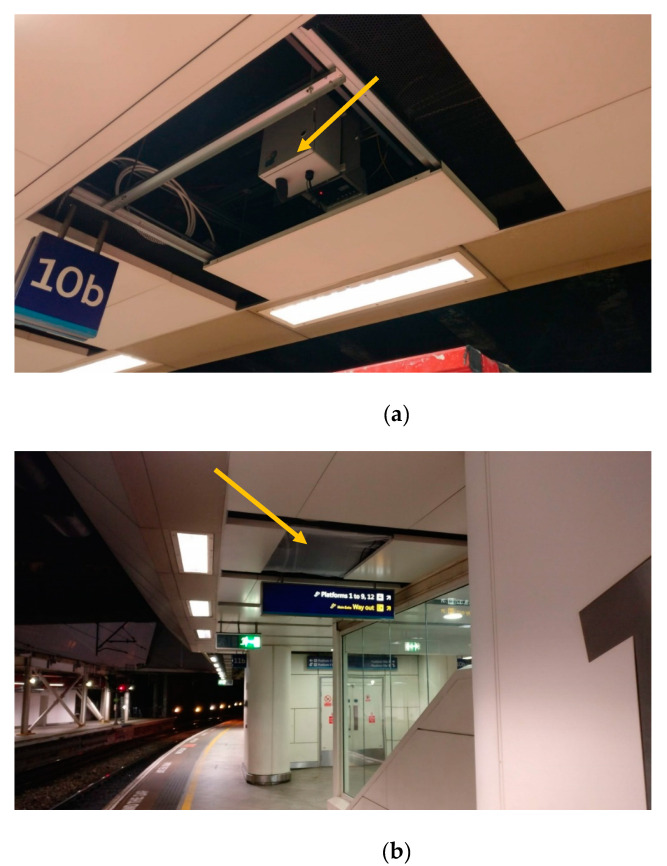

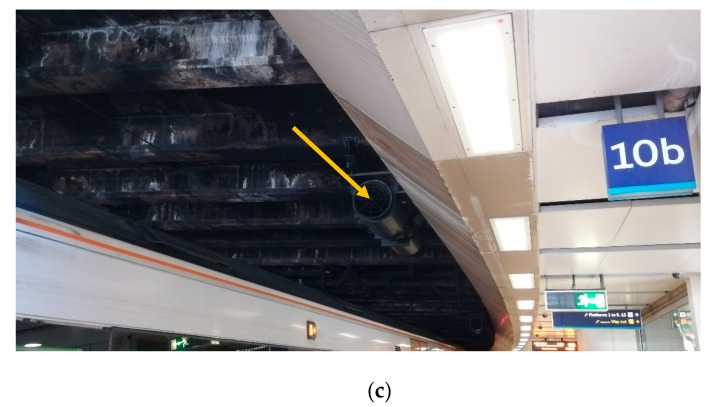
(**a**). Location of Praxis monitor on Platform 10B (photo: [12]). (**b**). Installation of Praxis monitor on Platform 11B in roof panelling of station (photo: [12]). (**c**). Example of one of 98 bi-directional impulse jet fans on Platform 10B installed above track level at 5 m (photo: M. Clegg, 2020).

Two South Coast Science Praxis monitors (South Coast Science, Brighton, UK), installed by Emission Solutions Ltd. (EMSOL) in January 2020, displayed in Figure 2a,b, sought to conduct measurements at this hotspot providing high temporal resolution data for both NO_2_ and PM. Their locations within the station on Platform 10B and 11B are represented by the blue triangle shown in Figure 1a.

Figure 1b shows the ground level concourse layout situated within a 2800 m^2^ dome atrium 25 m high and 50 m diameter [10]. At the East and West ends of the concourse level are stairs, escalators and lifts down to the platform level, which provide a limited exchange of air pollutants. This is an important source of air pollution given the high capacity for passengers at the concourse level coupled with a multitude of retail facilities situated within the atrium, both at concourse and above balcony level.

Figure 1c displays the geographical location of Birmingham New Street station within the centre of Birmingham. Much of the surrounding road network is low traffic volume due to the largely pedestrianised and commercial land-use of the city centre. The only notable exception to this is the A38 Queensway with traffic volumes of around 65,000 vehicles per day, based on annual average daily flow (AADF), of which 7000–10,000 are diesel powered buses, LGVs (Light Goods Vehicles), or HGVs (Heavy Goods Vehicles) [11]. In addition, many of the surrounding roads feature terminus stops for bus routes. The area within the inner ring road is now classified as a Clean Air Zone (CAZ) since June 2021. The significance of the ventilation of the air pollution from the station into the CAZ is the subject of further investigation by Birmingham City Council.

Air pollution sensors at Birmingham New Street have previously been mounted at 1.5–2.0 m above platform level to simulate height of mouth and nose for human respiration. The Praxis monitors utilised in this study were mounted within the roof panelling at 3 m above the platform (Figure 2a,b). This meant they were less disturbed by turbulence created by moving people and objects whilst providing an accurate assessment of ambient conditions on the respective platforms. The concrete roofing above the rails is 6 m above track level resulting in the fan system (shown in Figure 2c) to be at 5 m height.

Fixed monitor data for the pollutants NO_2,_ CO and particulates PM_1_, PM_2.5_, and PM_10_—atmospheric particles with aerodynamic diameter <1.0 µm (PM_1_) <2.5 µm (PM_2.5_) and <10 µm (PM_10_), respectively—were acquired from EMSOL for the period January to June 2020 [12]. Air quality was measured at two locations (Figure 1a), between the centres and West ends of Platform 10B and Platform 11B, respectively. Data were obtained from South Coast Science Praxis monitors placed in the roofing of the platforms (Figure 2a,b). The monitors utilise Alphasense electrochemical gas sensors of accuracy <±0.96 µg/m^3^ for NO_2_, <±0.6 µg/m^3^ for CO. For PM, the monitors utilise Alphasense optical particle counters (OPCs) capable of measuring particulates of aerodynamic diameter of 0.35 to 40 µm [13].

All sensors utilised in the Praxis monitors were operated within their optimal pollutant and environmental ranges minimising the possibility of erroneous readings. Furthermore, with the exception of NO_2,_ all the sensors were certified to be temporally stable by Alphasense preventing any drift in measurements or need for manual recalibration over the monitoring period. The sensors incorporate a temperature sensor to recalibrate microscale measurement deviations due to temperature change [14,15].

Before monitoring began all data were zero referenced by EMSOL following the manufacturer’s instructions. An acclimatisation period during January 2020 was established, which featured intermittent gaps within the data, and thus February 2020 was deemed the first month of reliable data. Data included continuous monitoring at a ten-second sampling rate from 1 February–30 June for Platform 10B and 1 February–25 April for Platform 11B. A 68-day monitoring period was established (1 February–8 April) for comparisons with the Hickman et al., 2018, study, which utilised a monitoring period of identical duration [2]. Ten-second interval data were utilised for analyses to provide high temporal resolution of short-term pollutant variability and to prevent loss of statistical precision when calculating short term averages (15 min STELs (short-term exposure limit) and EU (European Union) 1 h concentrations).

Unfortunately, due to COVID-19 restrictions, the UK was placed in a nationwide lockdown on 23 March 2020. This resulted in the final 15 days of the 68-day monitoring period being under different rail operating conditions. However, monitoring data from both platforms did allow assessment of pollution levels post lockdown and additionally the Platform 10B monitor, whose measurements continued until June 2020, provided data as restrictions were eased throughout May and June.

A 24 h TWA (Time Weighted Average) was established to discern daily pollutant conditions during the 68-day monitoring period and to allow comparison to the daily conditions measured under the previous fan system operation, during the Hickman et al., 2018 study [2]. From this, exceedance calculations for NO_2_, PM_2.5_, and PM_10_ could be determined to again compare to pre fan upgrade conditions. Subsequent TWAs of 8 h and 15 min were established to discern the current working conditions within the station in accordance with EH40/2005 WELs (Workplace Exposure Limits) and STELs (Short Term Exposure Limits).

## 3. Results

At a station such as Birmingham New Street, in Birmingham, UK, the station owner and the train operators, who have employees working there, have a legal duty, so far as is reasonably practicable, to manage the risks to health of their employees and users of the station (including passengers) from exposure to hazardous substances as specified by the Control of Substances Hazardous to Health Regulations 2002 (COSHH) (as amended) [16].

The results of the 68-day monitoring period displayed in Table 2 show a reduction in average NO_2_ 24 h concentrations of between 42% for platform 10b and 23% for platform 11b. Even so average NO_2_ concentrations of 224 µg/m^3^ and 293 µg/m^3^ at Platform 10B and 11B, respectively, are still of concern, given EU and Daily Air Quality Index (DAQI) Public Health 1 h regulations of 200 µg/m^3^ and Annual average of 40 µg/m^3^.

CO was measured well below EU 8 h limit values of 10,000 µg/m^3^ (listed as 10 mg/m^3^) at 307 µg/m^3^ and 457 µg/m^3^ on Platforms 10B and 11B, respectively. Maximal 24 h values followed the same trend of higher concentrations measured at Platform 11B than Platform 10B with 604 µg/m^3^ against 453 µg/m^3^.

**Figure 3:**
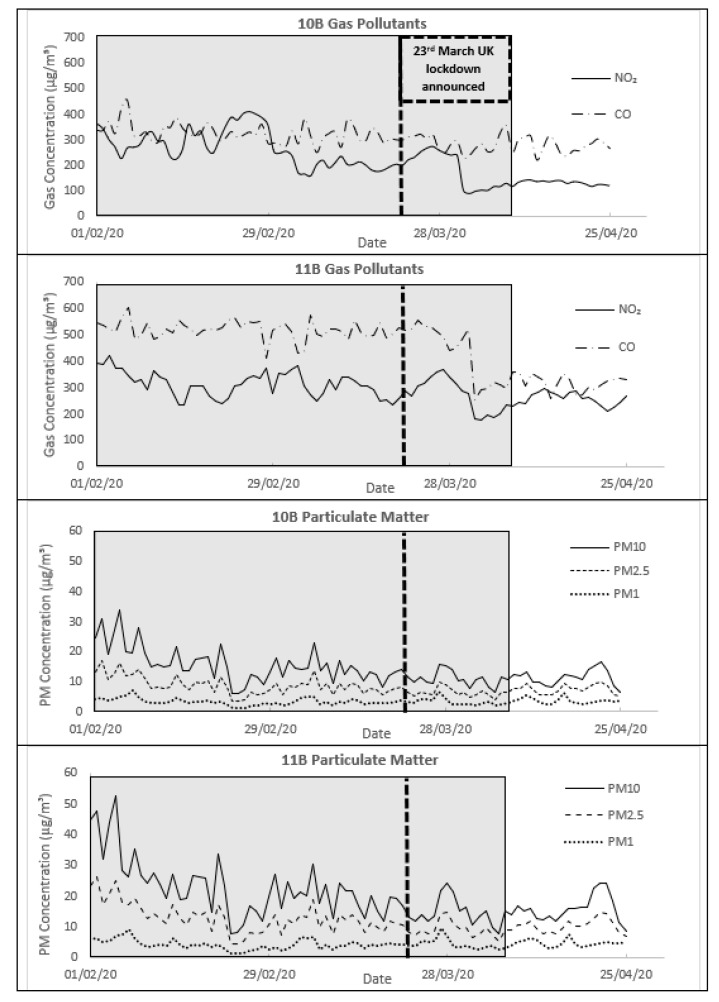
Time series of 24 h gaseous pollutants (NO_2_ and CO) and particulate matter (PM1, PM2.5, and PM10) concentrations between 1 February and 25 April 2020. The 68-day analysis period from 1 February to 8 April is marked in a grey shaded box. Note differing concentration scales for gaseous pollutants and PM.

Particulates followed the observed trend of higher concentrations at Platform 11B than at 10B with PM_10_ averages of 20 µg/m^3^ and 14 µg/m^3^, respectively. PM_2.5_ and PM_1_ also displayed the same observed trend with averages of 12 µg/m^3^ and 4 µg/m^3^ against 8 µg/m^3^ and 3 µg/m^3^. The maximal 24 h PM_10_ value measured was 52 µg/m^3^, which is in exceedance of the EU 50 µg/m^3^ air quality standard for ambient air, although this was only exceeded a total of 5 times between both platforms over the course of the 68-day monitoring period. Maximal 24 h PM_2.5_ concentrations were similar with 31 µg/m^3^ and 34 µg/m^3^ at Platform 10B and 11B, respectively. Finally, maximal PM_1_ concentrations were 11 µg/m^3^ and 17 µg/m^3^, respectively. As a whole, PM concentrations were at an order of magnitude lower scale than the gas pollutants and thus were plotted separately (Figure 3) to appropriately discern temporal variability. The size fractions of fine particulates (PM_1_ and PM_2.5_) from total particulates measured over the 68-day monitoring period were calculated at 57% at Platform 10B and 60% at Platform 11B. This suggested Diesel Engine Exhaust Emissions (DEEE) to be the likely source due to known fine particulate production from high temperatures and pressures in combustion processes [17,18]. The predominant small size fractions were of concern due to greater inhalation capacity of fine particles over coarse particles and their ability to reach deep into the human respiratory system [19,20] increasing risk of mortality [21,22,23,24]. For short-term exposures, such as passengers waiting for trains, this represents a minimal dosage; however, for operational staff within New Street, long-term daily exposure represents a potential increase in health risk. The plotted concentrations of all pollutants monitored are displayed in Figure 3.

Comparison of gas pollutants between Platform 10B and 11B showed similarity in peaks and troughs confirming interlinking of sources. The UK lockdown on 23 March showed no immediate effect with all gas pollutant concentrations remaining stable if not increasing in the case of NO_2_, consistent with concentrations measured at the Automatic Urban and Rural Network (AURN) stations (Figures S1–S3). However, a sharp drop in all pollutants occurred a week following the lockdown announcement due to a delayed reduction in train operations. This decrease represents a 1-week-delayed effect compared with the drop observed in mobility trends extracted from Apple Mobility Data (Figure S4) and CityMapper (Figure S5). The early stages of lockdown showed stable daily average gas pollutant concentrations until 19 April when concentrations began to rise.

Over the 68-day period, comparisons to the Hickman et al., 2018 study showed a minimum reduction in NO_2_ concentrations of 23% and maximum reduction of 42% [2]. For PM_10_ and PM_2.5_ concentrations a minimum reduction of 62% and maximum reduction of 81% (Table 2). The greatest reductions were found in all cases at Platform 11B. The reductions seen suggest that the fan system was effective, particularly at dispersing the solid particulates.

### 3.1. Exceedances of EU Regulations

**Figure 4:**
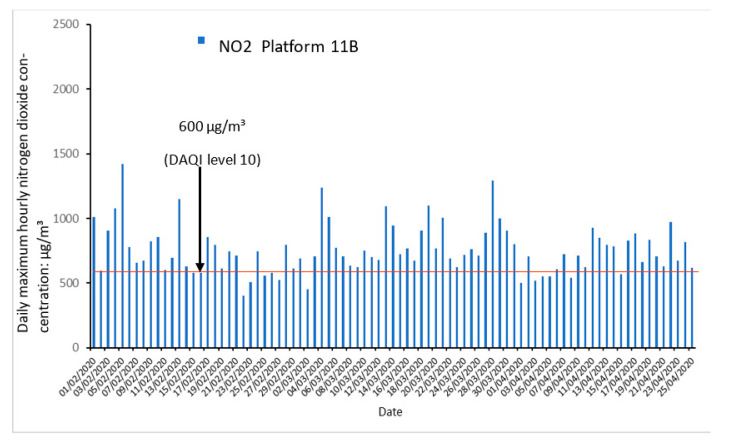
Retrospective assessment of the Daily Air Quality Index (DAQI) (73 out of 85 days (86%) exceeding DAQI level 10), for nitrogen dioxide concentrations in Birmingham New Street Station on Platform 11B.

During the 68-day period, the number of exceedances of the EU 1 h 200 µg/m^3^ NO_2_ limit value was calculated to be 1095 exceedances at Platform 10B and 1404 exceedances at Platform 11B. Despite both platforms experiencing more exceedances than the 1079 measured by the Hickman et al., 2018 study, it should be noted that maximal 1 h concentrations were significantly lower than conditions of the previous study. Previous maximal hourly concentrations were calculated at 2020 µg/m^3^ against 1422 µg/m^3^ at Platform 11B (30% reduction) measured in this study (Figure 4). In both this study and the Hickman et al., 2018 study, EU limits for NO_2_ were exceeded for the near entirety of the 05:00 to 00:00 passenger service operating hours due to widespread presence of DEEE within the station [2]. However, with such large reductions in maximal NO_2_ concentrations combined with the reductions in 68-day average NO_2_ concentrations, fan system effectiveness is strongly suggested. These concentrations also suggest New Street station is still a long way from maintaining accordance with the EU limit values; although, the station is not legally required to meet them.

Exceedances of the EU 24 h PM_10_ air quality standard of 50 µg/m^3^ showed large reductions from pre fan conditions with 9 exceedance days out of 68 on Platform 10B and 10 out of 68 on Platform 11B, versus 33 out of 68 days in the Hickman et al., 2018 study. Furthermore, the two other sites measured in the Hickman et al., 2018 study, at the East and West exposed ends of the platform area, reported 14 and 12 days, respectively, suggesting effective coarse particle dispersion by the new fan system. Maximal 24 h PM_10_ concentrations during the 68 days were 34 µg/m^3^ at Platform 10B and 53 µg/m^3^ at Platform 11B, which were again were lower than Hickman et al., 2018, maximal 24 h PM_10_ concentrations of 80–100 µg/m^3^ [2].

### 3.2. Occupational Exposure-Exceedances of WELs & STELs

WELs and STELs feature much higher pollutant concentration limit values for gas pollutants than EU air quality standards regulations stipulate. However, there are no limit values for any PM size fraction due to the heterogeneity of particulate composition.

**Figure 5:**
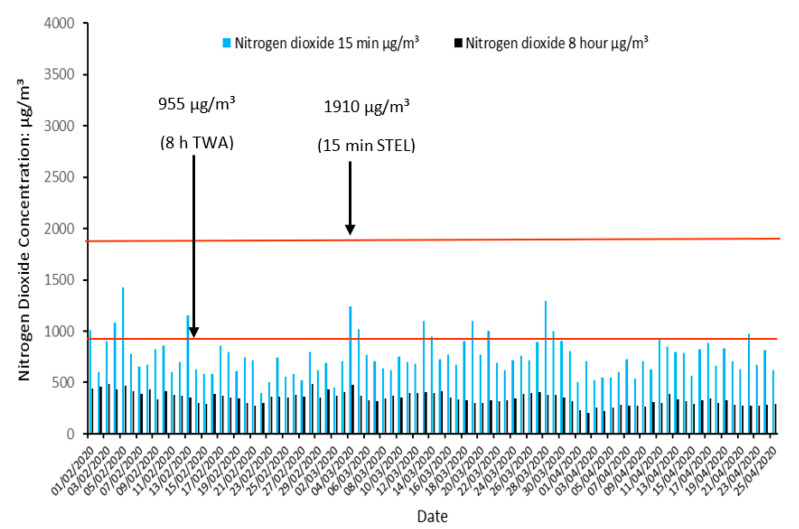
Retrospective assessment of nitrogen dioxide concentrations on Platform 10B in Birmingham New Street Station in comparison with the Workplace Exposure Limits.

In spite of the high concentrations of gas pollutants measured, and regular EU air quality standard regulation exceedances discussed previously, no gas pollutants exceeded limits from EH40/2005. NO_2_ came closest to exceeding its respective STEL value (1910 µg/m^3^) with maximal 15 min concentrations reaching 953 µg/m^3^ on Platform 10B (50% of STEL) and 1436 µg/m^3^ on Platform 11B (75% of STEL) (Figure 5). The NO_2_ WEL value of 955 µg/m^3^ was also not exceeded with the highest 8 h concentrations of 462 µg/m^3^ on Platform 10B (48% of WEL) and 488 µg/m^3^ on Platform 11B (51% of WEL).

Finally, CO exceeded neither the 117,000 µg/m^3^ STEL value nor the 23,000 µg/m^3^ WEL value throughout the entire study period.

### 3.3. Effect of COVID-19 on Concentrations of NO_2_, PM_2.5_, and PM_10_ at Platform 10B, New Street

**Figure 6:**
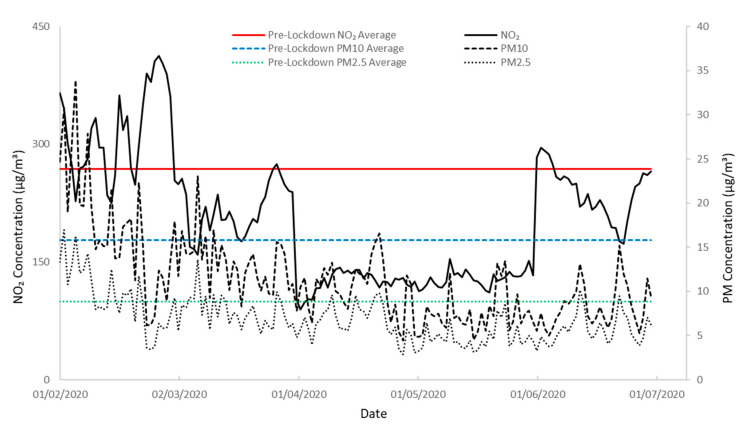
Dual *y*-axis timeseries plot of 24 h NO_2_ and PM_10_ and PM_2.5_ concentrations at Platform 10B from 1 February to 30 June 2020 with NO_2_ concentration on the left vertical axis whist PM concentration is on the right vertical axis. The horizontal solid, dashed, and stippled lines represent pre-lockdown average concentrations for the respective pollutants calculated between 1 February to 23 March 2020.

Figure 6 displays continuous 24 h concentrations of NO_2_, PM_10_, and PM_2.5_ from 1 February 2020, through the COVID-19 lockdown period of 23 March–11 May and into eased restrictions until the end of June 2020. Note the use of a dual *y*-axis due to the discrepancy in scale of gas pollutant concentrations against PM concentrations. As a reference, pre-pandemic average concentrations for each pollutant were plotted using 1 min data from 1 February to 23 March, which equalled 268 µg/m^3^ for NO_2_, 16 µg/m^3^ PM_10_, and 9 µg/m^3^ PM_2.5._

Despite lockdown announcement on the 23 March, it took a week for the train timetable to be reduced and concentrations of all pollutants increased continually over the course of a week to near pre-COVID-19 average values, consistent with concentrations measured at ambient air AURN stations (Figures S1–S3). However, a week delay until 30 March, saw values level off before sharply decreasing to concentrations less than half of pre-COVID-19 averages. Graphically, the resultant effect was a two-month trough in pollutant concentration data from 31 March to 31 May. NO_2_ concentrations averaged 135 µg/m^3^ during this period whilst PM_10_ and PM_2.5_ averaged 9 µg/m^3^ and 5 µg/m^3^, respectively. These averages equated to 50%, 56%, and 55% of the pre-lockdown averages. The easing of lockdown restrictions on 11 May enabled journeys previously deemed non-essential during lockdown. This appeared to have little immediate effect on pollutant concentrations with values staying within 15% of averages over April and May, consistent with the mobility trends reported by Apple Mobility and CityMapper in Birmingham (Figures S4 and S5). However, at the beginning of June, NO_2_ concentrations rebounded in excess of the pre-lockdown average with a 24 h concentration of 283 µg/m^3^ on the 1 June. This represented a doubling within a single day; nevertheless, it took six days for the corresponding increase in PM concentrations to be observed.

Overall, the COVID-19 pandemic’s effect on rail operations at Birmingham New Street station produced a distinct 50–56% improvement in air quality. This provides a pointer to the level of potential benefits that could be made by reducing dependency on DEEE producing trains.

## 4. Conclusions

The fan system upgraded at Birmingham New Street station resulted in a 23–42% reduction in NO_2_ and significant reduction in PM when compared to the Hickman et al., 2018, study, and thus the intervention is deemed effective [25]. TWAs for all pollutants were successfully calculated allowing exceedances to be analysed for EU regulations, WELs and STELs. Concentrations of NO_2_ were still found to regularly exceed EU air quality standards, which are still a health risk for workers and passengers. In contrast, WEL and STEL for NO_2_ were met during the monitoring campaign. Additionally, the effect of the COVID-19 pandemic on air quality within New Street was found to reduce pollutant concentrations by a further 50% for NO_2_, 56% for PM_10_, and 55% for PM_2.5_.

The Centre for Cities calculated that NO_2_ levels in Birmingham’s city streets were reduced by about 37% and PM_2.5_ by about 23% due to the first lockdown [26]. The reductions were not immediate after UK Government lockdown restriction announcements seen by the delay observed before pollutants decreased. This study supports claims by previous studies [8] that the most effective method of mitigating high concentrations of both gas pollutants and PM within New Street would be the replacement of diesel combustion powered trains with electric or diesel/electric hybrid systems. The latter would not only allow the prevention of intense DEEE emissions at Birmingham New Street station, but would also have the capability of operating on all areas of the UK rail network, including those that are not currently electrified.

Further monitoring and research are needed at New Street station to follow up on the results of this research to examine the impact of ventilating the station’s NO_2_ and PM into the middle of the Birmingham Clean Air Zone (CAZ), which has only been in operation since June 2021. 

## Figures and Tables

**Table 1:** Workplace Exposure Limits (WELS) and EU Air Quality Standards (EUAQS).

Pollutant	WELS Occupational Health	STELS Occupational Health	EUAQS Public Health	EUAQS Public Health
	8 h	15 min	1 year	Short Term
NO_2_	955 µg/m^3^	1910 µg/m^3^	40 µg/m^3^	200 µg/m^3^ 1 h mean
PM_10_	-	-	40 µg/m^3^	50 µg/m^3^ 24 h mean
PM_2.5_	-	-	25 µg/m^3^	-
CO	23 mg/m^3^	117 mg/m^3^	-	10 mg/m^3^ 8 h mean

**Table 2:** Platform 10B and 11B average and maximal 24 h pollutant concentrations for the 68 day monitoring period (1 February–8 April 2020) In comparison to Hickman et al., 2018. All pollutant concentrations measured in µg/m^3^.

Platform	NO_2_	CO	PM_1_	PM_2.5_	PM_10_
Average 24 h Hickman 2018 (µg/m^3^)	383	-	-	42	53
10B Average 24 h Concentration (µg/m^3^)	224	307	3	8	14
% Reduction	42%			81%	74%
10B Max 24 h Concentration (µg/m^3^)	413	453	11	31	34
11B Average 24 h Concentration (µg/m^3^)	293	457	4	12	20
**% Reduction**	23%			71%	62%
11B Max 24 h Concentration (µg/m^3^)	422	604	17	34	53

## Data Availability

Restrictions apply to the availability of these data. Data was obtained from EMSOL and are available with the permission of EMSOL at https://emsol.io (accessed on 15 July 2020).

## Supplementary Materials

The following are available online at https://www.mdpi.com/article/10.3390/ijerph19010575/s1, Figure S1: NO_2_ (µg/m^3^) concentrations measured at Birmingham A4540 Roadside AURN station from 1 February 2020 to 30 June 2020. Figure S2: PM_10_ (µg/m^3^) concentrations measured at Birmingham A4540 Roadside AURN station from 1 February 2020 to 30 June 2020. Figure S3: PM_2.5_ (µg/m^3^) concentrations measured at Birmingham A4540 Roadside AURN station from 1 February 2020 to 30 June 2020. Figure S4: Apple Mobility Trends in Birmingham (UK) from 1 February 2020 to 30 June 2020. Figure S5: CityMapper Mobility Index in Birmingham (UK) from 1 February 2020 to 30 June 2020.
