# Supplementary material for: Intervention of an Upgraded Ventilation System and Effects of the COVID-19 Lockdown on Air Quality at Birmingham New Street Railway Station
Source: Int J Environ Res Public Health. 2022 Jan 5;19(1):575. doi: 10.3390/ijerph19010575 (PMC8744564; doi:10.3390/ijerph19010575)
Supplement: Supplementary file 1 [file ijerph-19-00575-s001.zip › ijerph-1501998-supplementary.pdf]

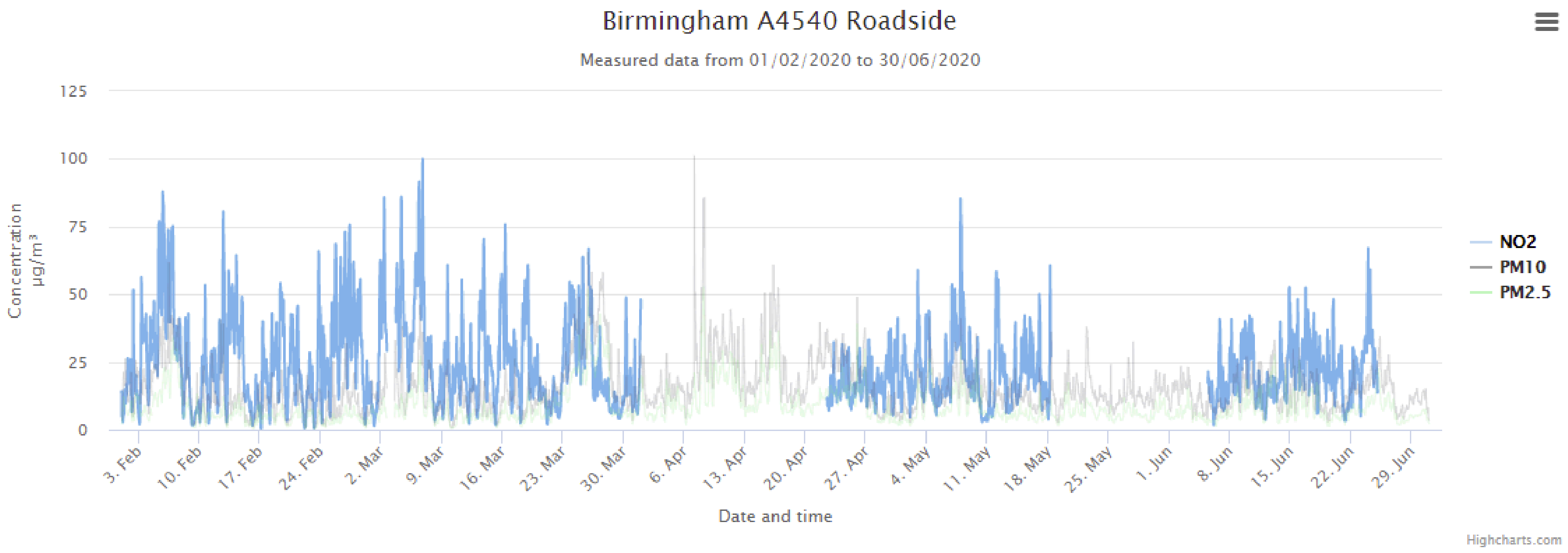

Figure S1. NO_2 ($\mu g/m^3$) concentrations measured at Birmingham A4540 Roadside AURN station from 01/02/2020 to 30/06/2020.

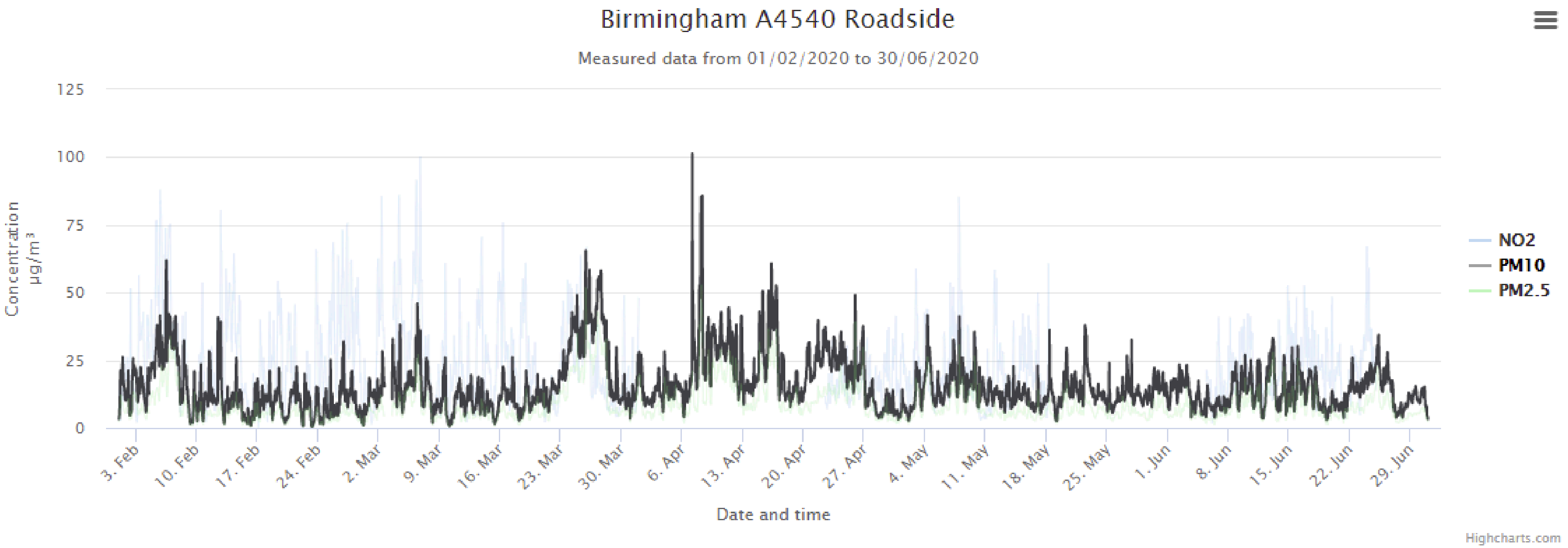

Figure S2. PM_{10} ($\mu g/m^3$) concentrations measured at Birmingham A4540 Roadside AURN station from 01/02/2020 to 30/06/2020.

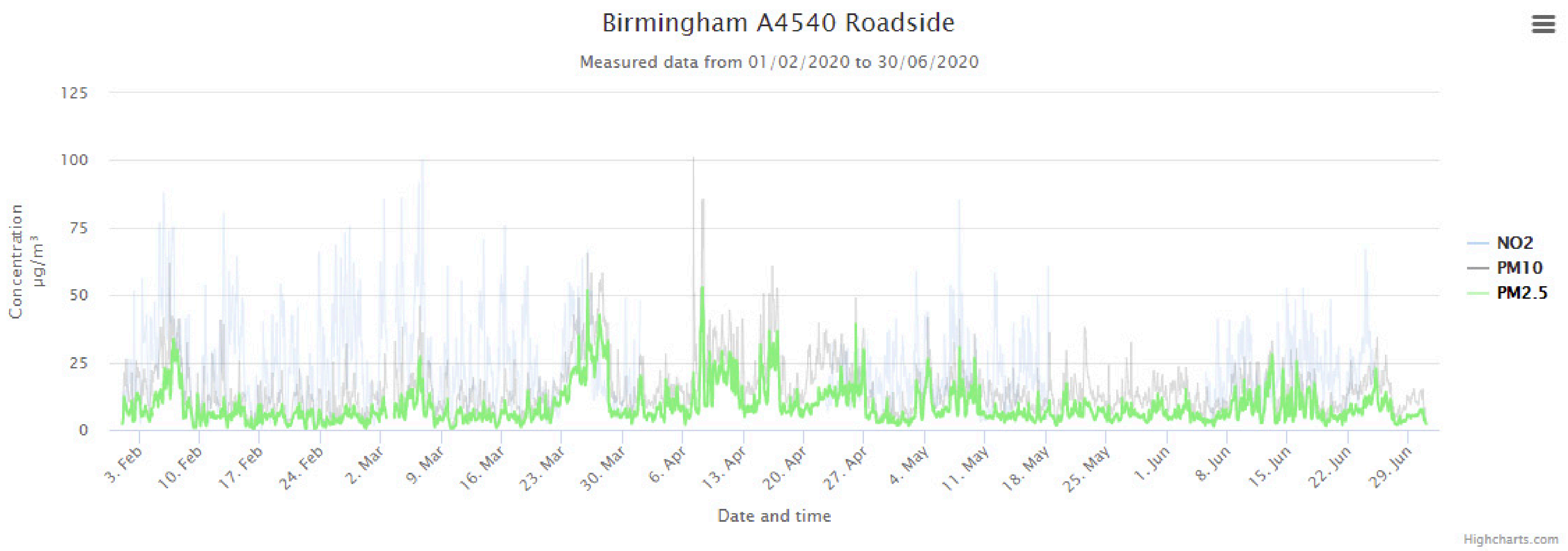

Figure S3. $PM_{2.5}$ ($\mu g/m^3$) concentrations measured at Birmingham A4540 Roadside AURN station from 01/02/2020 to 30/06/2020.

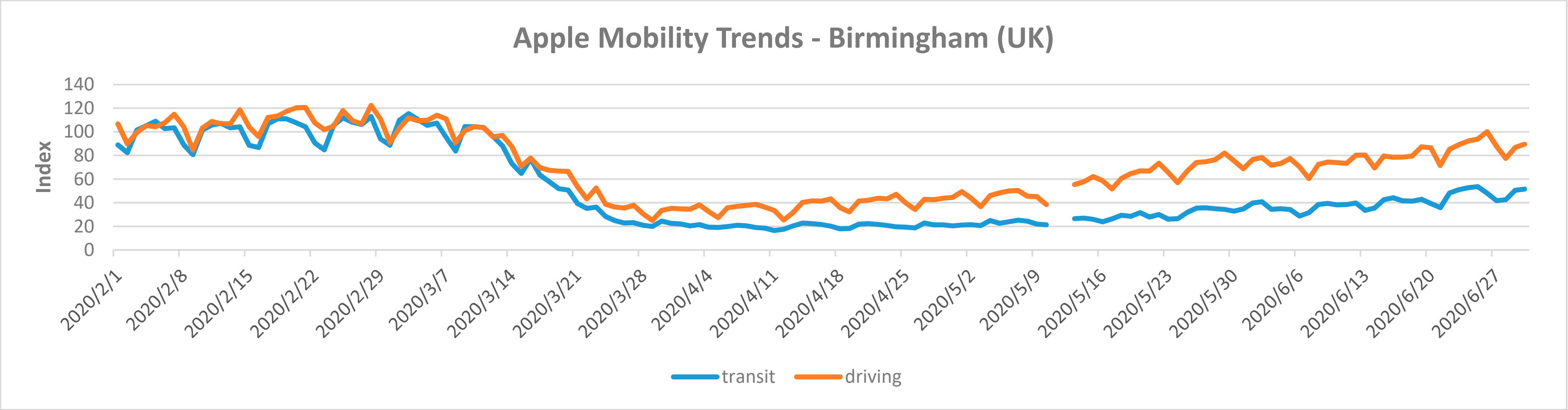

Figure S4. Apple Mobility Trends in Birmingham (UK) from 01/02/2020 to 30/06/2020.

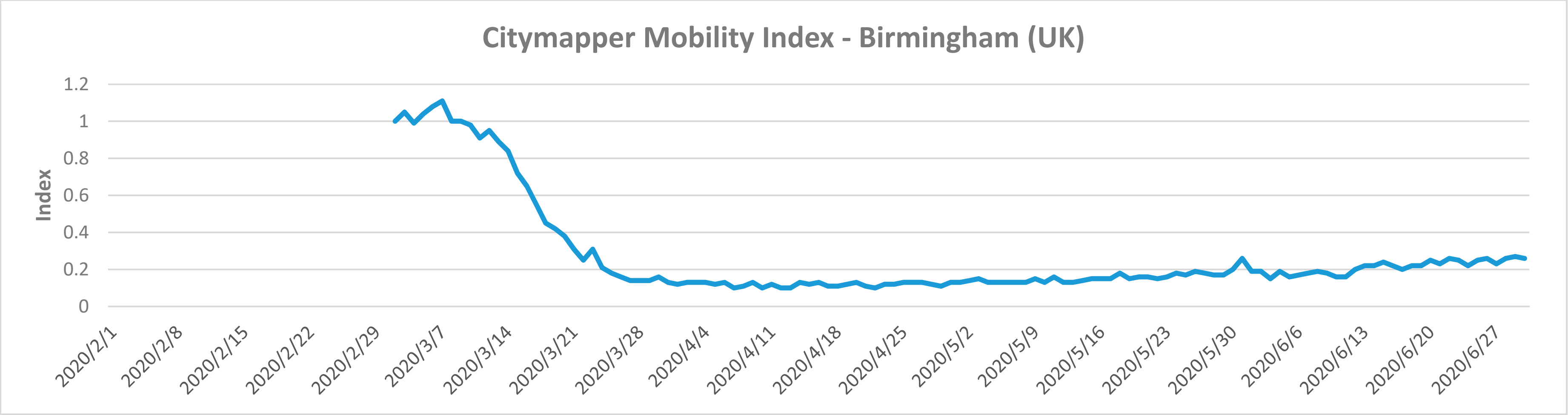

Figure S5. CityMapper Mobility Index in Birmingham (UK) from 01/02/2020 to 30/06/2020.
